# Comparison of surgical resection and radiofrequency ablation for stages I and II elderly hepatocellular carcinoma patients (≥ 65 years): A SEER population-based propensity score matching’s study

**DOI:** 10.3389/fonc.2022.903231

**Published:** 2022-08-25

**Authors:** Qingqing Xie, Yongwen Yang, Bin Qu, Ping Xiao, Faqing Tang, Haoming Shen

**Affiliations:** ^1^ Department of Clinical Laboratory, Third Affiliated Hospital of Guangxi University of Chinese Medicine, Liuzhou, China; ^2^ Department of Clinical Laboratory, Xiangya Hospital, Central South University, Changsha, China; ^3^ Department of Clinical Laboratory, Hunan Cancer Hospital and The Affiliated Cancer Hospital of Xiangya School of Medicine, Central South University, Changsha, China

**Keywords:** hepatocellular carcinoma, competitive risk model, propensity score matching, conditional survival probability, surgery

## Abstract

**Objectives:**

The treatment for hepatocellular carcinoma (HCC) remains controversial and limited in elderly patients. Therefore, we aimed to explore treatment choices for the elderly patients (≥ 65years) following surgical resection (SR) versus radiofrequency ablation (RFA) with HCC (single lesion less than 5 cm).

**Methods:**

We used SEER database to identify HCC patients who received treatment of SR/RFA. Kaplan–Meier method and Cox proportional hazards regression method were used to determine the prognostic factors associated with overall survival (OS) and disease-specific survival (DSS). In addition, RFA group and SR group patients were matched with 1:1 propensity score matching (PSM) for diagnosis age, sex, race, marital, American Joint Committee on Cancer (AJCC), grade, radiotherapy, and chemotherapy to decrease the possibility of selection bias. Conditional disease-specific survival (CS) was estimated using the life-table method.

**Results:**

A total of 794 patients who underwent SR and 811 patients who underwent RFA were confirmed from the SEER database. Surgery type was an independent risk factor for HCC. Survival analysis indicated that SR, races, AJCC I, no chemotherapy treatment, and grade I were cumulative risk factors that can significantly improve median survival for HCC (*P* < 0.05). After PSM analysis, only surgery type was significantly improved median survival of HCC patients (SR vs. RFA, HR: 0.644, 95% CI: 0.482–0.86; *P* < 0.001). For RFA group, the 2-, 3-, and 5-year CS rates were approximately 71%, 65%, and 62%, respectively, and corresponding to 82%, 80%, and 78% in the SR group.

**Conclusion:**

SR treatment can provide survival benefits for elderly patients of <5 cm single lesion HCC.

## Introduction

Hepatocellular carcinoma (HCC) is the most common primary malignancy of the liver with high mortality rates in the world, and more than 850,000 patients were newly diagnosed each year; among these new cases, about 50% of patients were aged 65 years or older (1). Tumor stage is the strongest prognostic indicator in HCC patients, with curative treatments only available for patients with early stage HCC. Patients who satisfy the Milan criteria (2) (single lesion less than 5 cm) are optimal candidates for liver transplantation (LT), but high costs, donor shortage, and lifelong immunosuppression often limits LT (3), and surgery resection (SR) is still the first-line treatment for primary HCC. However, less than 20% of HCC patients receive radical SR. For HCC patients who are unsuitable for SR or LT, the best choice is radiofrequency ablation (RFA) (4), but many studies have revealed that RFA is only comparable with hepatic resection in terms of long-term survival for HCC patients with single lesion less than 3cm (5–7). It remains unclear which approach is more beneficial for patients with single nodular HCC < 5 cm.

Whether SR or RFA is a better treatment for elderly HCC patients has previously been debated. Some RCT results published so far are contradictory (8, 9). RFA and hepatectomy had similar survival rates in both trials, whereas RFA was inferior to resection in terms of patient survival and tumor recurrence in the other trials (10). Some studies have shown that the therapeutic effect of RFA is comparable with that of surgical resection (SR) for small HCCs with a diameter of less than 3 cm (11). Some RCTs of randomized trials found that SR was associated with better overall and disease-free survival compared with RFA (8, 12, 13). However, SR resulted in more treatment-related complications and a longer hospital stay than RFA (12). A disadvantage of these studies is that they are highly variable with a relatively short median follow-up period, varying degrees of protocol violations, and the number of patients who were lost to follow up (14). More importantly, no study has yet compared the efficacy of surgical treatment with RFA for solitary HCC with T1 or T2 metastasis, especially in elderly patients (over 65 years old), considering different tumor sizes.

Aging is a major risk factor and poor prognostic factor for most chronic diseases. The incidence of HCC increased after the age of 40 years, and it was estimated that the incidence of HCC will increase by approximately 59% by 2030, more than 50% of which will be in people aged 65 years or above (15, 16). Compared with the younger population, complications such as diabetes, poor cardiopulmonary function, and renal insufficiency are common for elder adults (aged ≥65 years), which leading to higher severity and poorer prognosis after SR or RFA (11, 17, 18). Therefore, a more careful evaluation of the risk-benefit ratio in terms of SR and RFA treatment is required.

In this study, we aimed to investigate the outcomes of SR versus RFA in elderly patients with first diagnosed stages I and II HCC (single nodular HCCs ≤ 5 cm) using the population-based SEER registry, which will provide scientific evidence for clinicians in the selection of treatment strategies for this population.

## Materials and methods

### Data sources

The SEER database is sponsored by the National Cancer Institute and aggregates data from 18 cancer registries (https://seer.cancer.gov/). SEER is a collection of population based on cancer registries covering approximately 28% of the U.S. population. The SEER database contains information about patient demographics and cancer characteristics, such as sex, age at diagnosis, year of diagnosis, race, marital status, tumor grade and stage, histological type, treatment, and patient survival time. We also decrypt the session data that are encrypted in the database and reproduce individual data in SEER*Stat Database.

### Inclusion and exclusion criteria

For accurate enough survival information, this research extracted patient data diagnosed from 2010 to 2015 and cause of death information was available until 31 December 2017. Patients with HCCs were selected (8170/3: ICD-O-3) and selected tumor cases from the primary site of the HCC. The study included only patients with American Joint Committee on Cancer (AJCC) T1/2 patients with different treatment SR versus RFA. Only patients above 65 years of age were included. Patients with any of the following criteria were also excluded: multiple lesions, not the first malignant tumor, survival less than 3 months, and tumor metastasis. Finally, 1,605 eligible patients diagnosed with HCC remained.

### Variable selection

Definition and information about the variables can be found in the SEER database. We gathered variables of age, race, and marital status. AJCC tumor node metastasis (TNM) stage, AJCC N status, AJCCM status, grade, surgical therapy, radiotherapy, chemotherapy, causes of death, and survival months from the SEER database. Age was classified as 65–70 and 71 years old. For race classification, we consider three categories (white, black, and others). Marital status was classified as unmarried/married/divorce/separation and widowed/other. AJCC TNM stage was classified as stage I or II. AJCC T status was classified as T1 or T2. Histologic grade was classified as grades I, II, III, IV, and unknown. Chemotherapy treatment and radiotherapy were all defined as receiving corresponding therapy or not. Surgery type was classified as RFA or SR.

### Statistical analysis

Data analyses were performed using R Statistical software (R4.1.1, survival packages, forestplot packages, cmprsk packages, tidyverse packages, and ggplot2). The Kaplan–Meier curve was used to estimate the overall survival (OS) and disease-specific survival (DSS) in different groups, and the differences between the curves were analyzed by log-rank test. Univariate and multivariate Cox regression models were performed to estimate the hazard ratios(HRs)and 95% confidence interval (CI) to analyze the independent prognostic factors associated with OS and DSS in HCC patients. Cumulative incidence function (CIF) and proportional subdistribution hazard model were adopted to explore risk factors for HCC-specific death (HCSD) and other cause-specific death (OCSD). For the competing risk model, HCSD and OCSD were two competing endpoint events. First, CIF was calculated, as well as CIF grouped by age, race, marital status, grade, AJCC stage, T status, N status, surgery, radiotherapy, or chemotherapy. We plotted CIF curves for every variable and performed the Gray’s test to recognize differences for two events in the CIF. Second, the propensity score matching (PSM) method was employed because of substantial differences that exist in terms of clinical characteristics between patients with RFA or SR. Nearest-neighbor matching in PSM is a method that can be used for causal inference when more confounding factors are involved in non-randomized controlled studies. We used the MatchIt package, then set the caliper value to 0.1 based on the obtained propensity values and considering the number of samples and the quality of pairs, and finally matched the RFA group with the SR group patients in a 1:1 ratio. Third, for multivariate competing risk survival analysis, we constructed the Fine and Gray proportional sub-distribution hazard model to predict HCSD and OCSD by R package of cmprsk. Fourth, conditional disease-specific survival (CS), the origin of which is conditional probability in biostatistics, can be calculated using the life-table method. The 5-year conditional disease-specific survival (CS5) at x years indicates the likelihood of an additional 5-year survivorship for a survivor who has already survived for x years after the initial treatment, calculated as follows: CS5 = DSS (x + 5)/DSS(x). Initial prognostic estimates for patients were usually based on individual characteristics after surgery. CS estimates were recalculated by incorporating the clinicopathological characteristics and the survival time. CS analysis was employed to assess possible changes in the prognostic impact of the aforementioned factors over time after resection. *P* < 0.05 was significant, and *P* values were two sided.

## Results

### Patient characteristics

**Figure 1:**
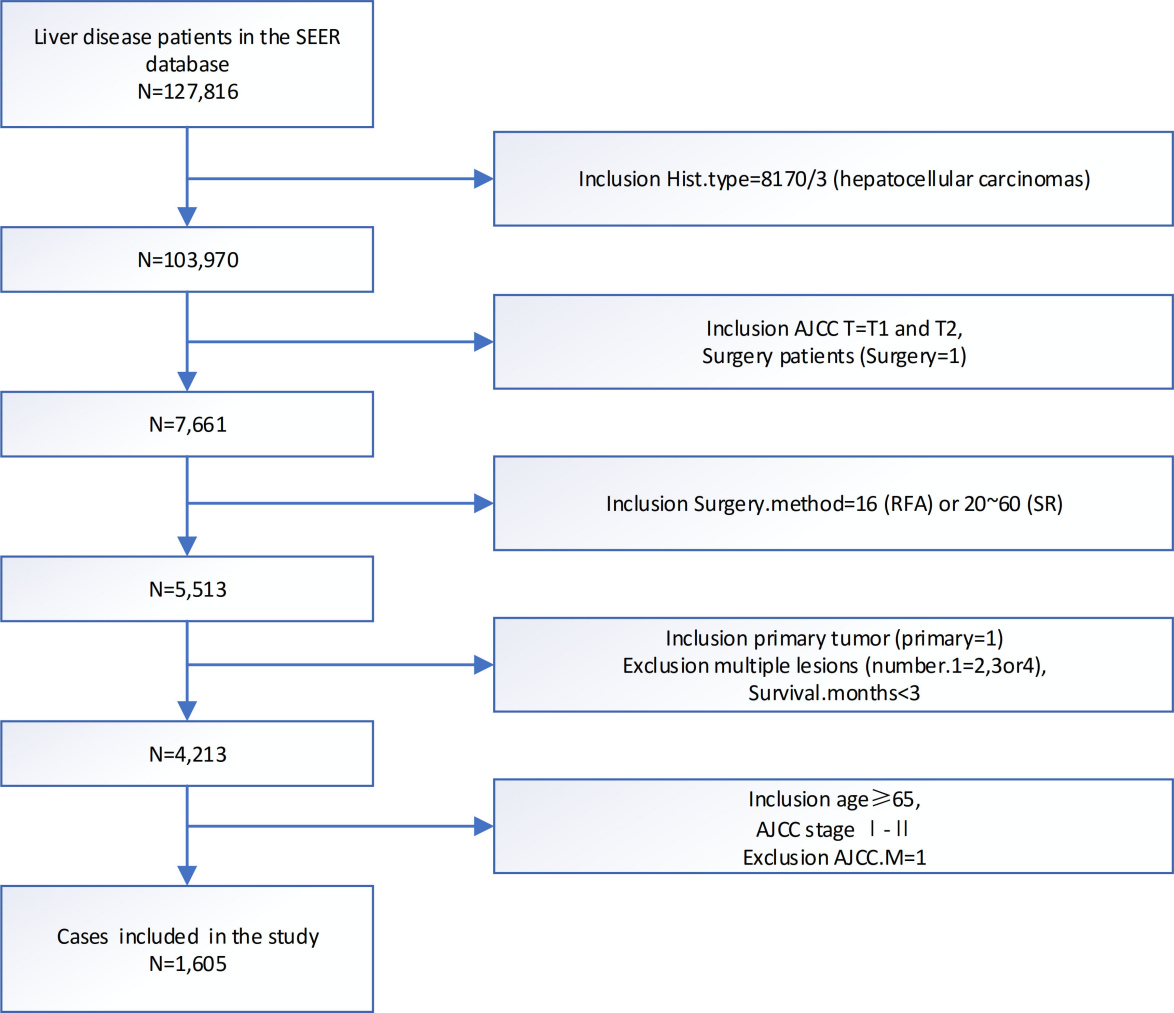
Flowchart of patients identified in this study. SEER, end results; N, number; AJCC, American Joint Committee on Cancer; TNM, tumor node metastasis.

As **Figure 1** showed, we originally included 103,970 patients from the SEER database. Subsequently, we excluded 80,335 patients with T3 or T4 stage, 15,947 no surgery patients, 2,148 patients with other surgery, and 858 patients who were not primary tumors. Finally, we identified 1,605 eligible patients diagnosed with elderly patients first diagnose for < 5 cm single lesion T1/2 HCC remained.

Baseline information of the included patients was shown in **Table 1**. Most patients were men (1,086, 68%), White race (973, 61%), and married (938, 58%). There were 1,201 (75%) patients with AJCC I and 404 (25%) patients with AJCC II. As for grading and staging system, there were more patients stayed as grade II (579, 36%) and T1 status (1,201, 75%). Of all patients who chose SR or SFA, 79% (1,270) had no chemotherapy and 97.8% (1,569) had no radiation.

**Table 1:** Baseline characteristic of the included primary HCC patients in the SEER database.

Variables	Total (*n* = 1605)	RFA (*n* = 811)	SR (*n* = 794)	*p*
Age.cat, *n* (%)				0.346
~70	768 (48)	398 (49)	370 (47)	
71~	837 (52)	413 (51)	424 (53)	
Sex, *n* (%)				0.575
Female	519 (32)	268 (33)	251 (32)	
Male	1086 (68)	543 (67)	543 (68)	
Race, *n* (%)				0.037
Black	146 (9)	76 (9)	70 (9)	
Others	486 (30)	222 (27)	264 (33)	
White	973 (61)	513 (63)	460 (58)	
Marital, *n* (%)				0.128
Divorced/Separated	184 (11)	99 (12)	85 (11)	
Married	938 (58)	452 (56)	486 (61)	
Single/Unmarried	185 (12)	95 (12)	90 (11)	
Widowed/Others	298 (19)	165 (20)	133 (17)	
Diagnosis, *n* (%)				0.112
2010	198 (12)	88 (11)	110 (14)	
2011	219 (14)	112 (14)	107 (13)	
2012	232 (14)	120 (15)	112 (14)	
2013	276 (17)	158 (19)	118 (15)	
2014	308 (19)	148 (18)	160 (20)	
2015	372 (23)	185 (23)	187 (24)	
AJCC, *n* (%)				0.594
I	1201 (75)	612 (75)	589 (74)	
II	404 (25)	199 (25)	205 (26)	
AJCC.T, *n* (%)				0.594
T1	1201 (75)	612 (75)	589 (74)	
T2	404 (25)	199 (25)	205 (26)	
Chemotherapy, *n* (%)				< 0.001
NO	1270 (79)	559 (69)	711 (90)	
YES	335 (21)	252 (31)	83 (10)	
Grade, *n* (%)				< 0.001
I	272 (17)	120 (15)	152 (19)	
II	579 (36)	154 (19)	425 (54)	
III–IV	188 (12)	31 (4)	157 (20)	
Unknown	566 (35)	506 (62)	60 (8)	
Radiotherapy (%)				1
NO	1569 (97.8)	793 (97.8)	776 (97.7)	
YES	36 (2.2)	18 (2.2)	18 (2.3)	
Surgery Type, *n* (%)				< 0.001
RFA	811 (51)	811 (100)	0 (0)	
SR	794 (49)	0 (0)	794 (100)	

### Survival analysis in OS and DSS before PSM

**Figure 2:**
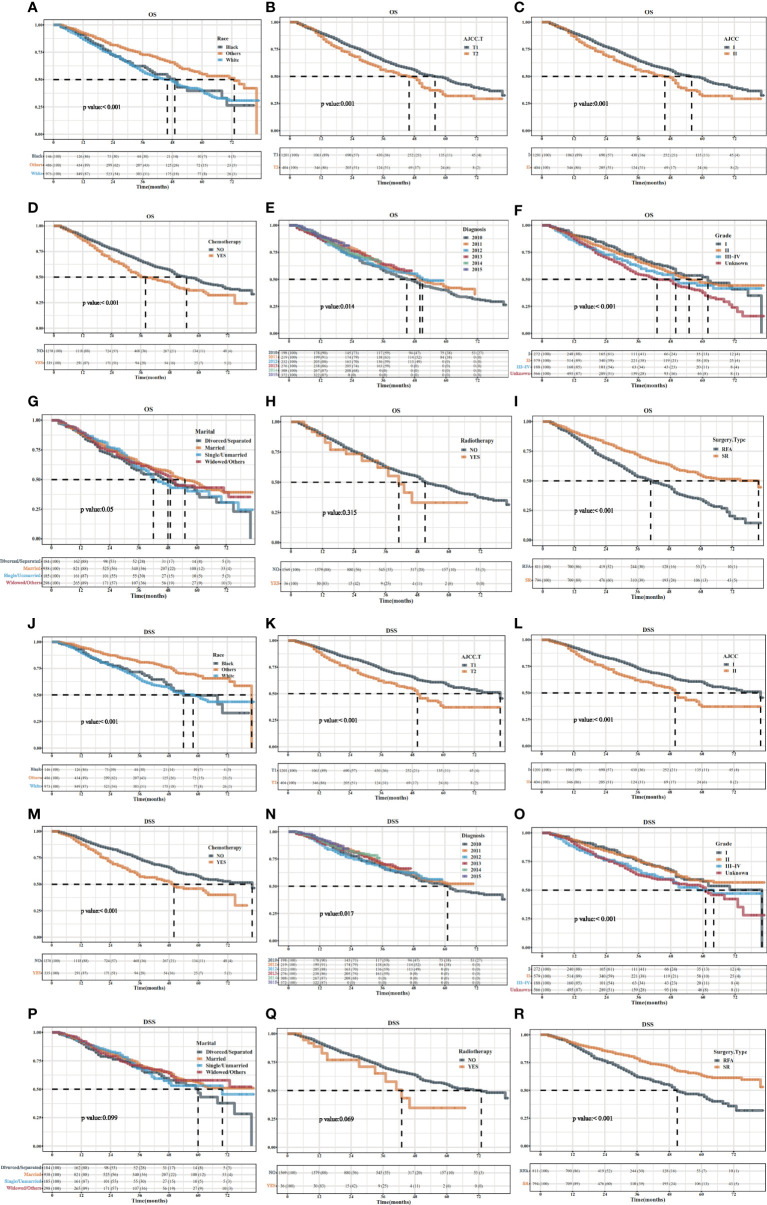
The OS and DSS curves in HCC patients with SR or SFA before PSM. The OS/DSS curves in HCC patients of different race **(A/J)**, AJCC T1/2 **(B/K)**, AJCC 1/2**(C/L)**, chemotherapy**(D/M)**, diagnosis**(E/N)**, grade**(F/O)**, marital**(G/P)**, radiotherapy**(H/Q)** group with SR or SFA before PSM. **(I/R)** The OS/DSS curves in HCC patients with SR or SFA before PSM.

In general, the patients with HCC T1/2 in SR group had a longer OS (**Figure 2I**) and DSS (**Figure 2R**) than that in the RFA group showed by Kaplan–Meier analysis before PSM (*P* < 0.001). Other races, no chemotherapy, AJCC1/2, AJCC T1/2, AJCC Grade I, diagnosis years, or chemotherapy or not had longest median OS. Similar results were observed for (*p* < 0.05, **Figures 2A–F, J–O**). There were no statistically significant differences in median survival among patients with marital status or with or without radiation therapy (*p* > 0.05, **Figures 2G, H, N, P, Q**).

### Univariate and multivariable Cox proportional hazard models

**Figure 3:**
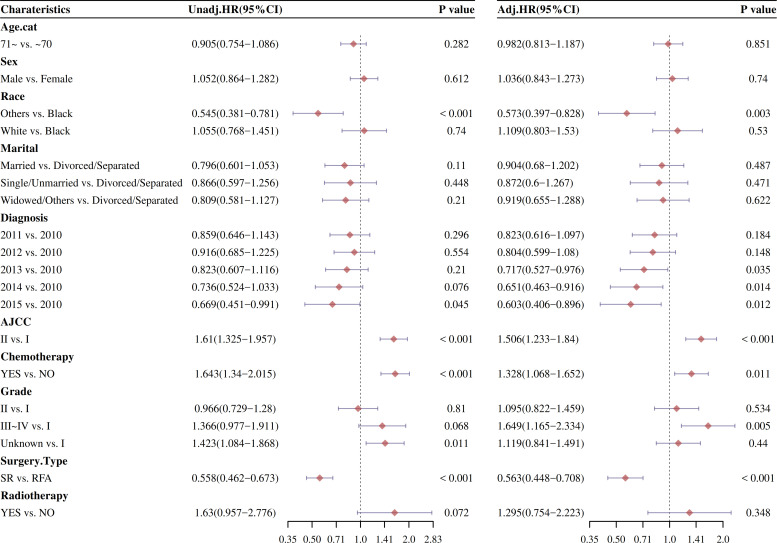
OS and DSS were analyzed by the univariate Cox proportional hazard models before PSM.

Afterward, the univariate and multivariable Cox analysis of factors affecting OS and DSS was analyzed. As showed in **Figure 3**, the univariate analysis showed that surgery type was significantly examined to be a surely prognostic factor (HR: 0.54, 95% CI: 0.459–0.635; *P* < 0.001). The patients who not received chemotherapy had better results than those who received chemotherapy (HR: 1.445, 95% CI: 1.205–1.732; *P* < 0.001). Other races, AJCC I, grade I, AJCC I, and 2015 diagnosis with HCC were individually associated with improved survival (*P* < 0.05). Similar outcomes were obtained from the univariate analysis of DSS.

The effect of stage and treatment modality on OS and DSS was further examined in multivariable Cox proportional hazard models before PSM. The multivariable Cox analysis also showed that surgery type was significantly checked to be a prognostic factor (*P* < 0.001). Other races, AJCC I, grade I, and 2013/2014/2015 diagnosis with HCC were also individually associated with improved survival (*P* < 0.05).

### Univariate analysis by CIF before PSM

**Figure 4:**
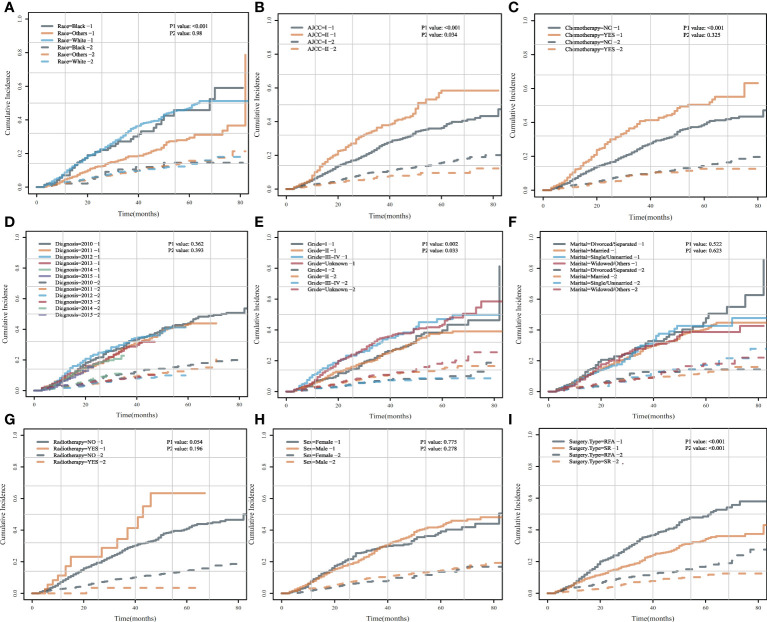
Cumulative incidence curves for each characteristic before PSM. **(A)** HCSD/OCSD cumulative incidence curves in HCC patients of different races **(A)**, AJCC **(B)**, chemotherapy **(C)**, diagnosis **(D)**, grade **(E)**, marital **(F)**, radiotherapy **(G)**, gender **(H)**, surgery type **(I)** group with SR or SFA before PSM.

To further explore the risk factors of HCSD/OCSD, we carried out univariate analysis by CIF on all the risk factors. CIF curves for all variables were shown in **Figure 4**. SR was a cumulative risk factor that significantly increased median HCSD/OCSD survival (*P* < 0.05). We also found that other races, AJCC I, no chemotherapy treatment, and grade 1 were also cumulative risk factors that can significantly improve median for HCSD (*P* < 0.05).

### Univariate and multivariate analysis by fine and gray model

**Figure 5:**
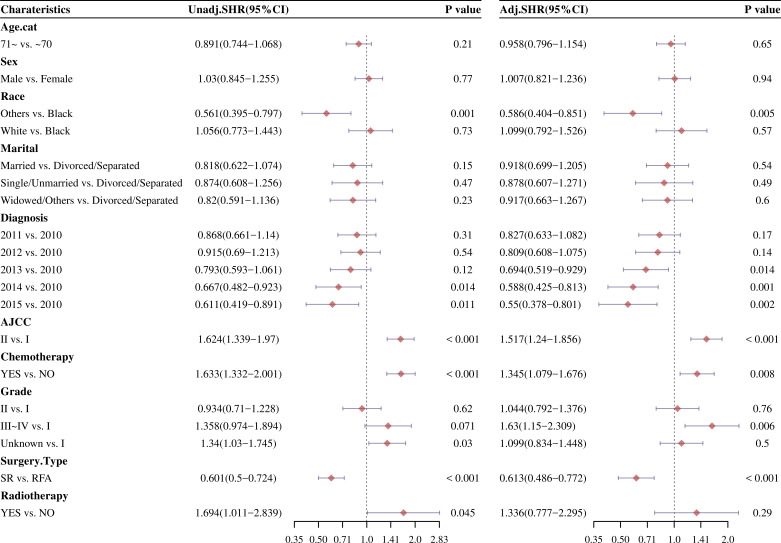
Forestplot of OS and DSS were analyzed by the univariate Cox proportional hazard models after CIF analysis before PSM.

The competitive risk model is constructed by using CIF method, and the advantages of univariate and multivariate COX regression after model construction are compared with those without model construction. After CIF risk competition model calculation, the univariate and multivariable Cox analysis of factors affecting OS and DSS was analyzed. As showed in **Figure 5**, the univariate analysis manifested that surgery type was significantly considered to be a positive cumulative incidence factor (HR: 0.601, 95% CI: 0.5–0.724; *P* < 0.001). The patients who not received chemotherapy had better results than those who received chemotherapy (HR: 1.633, 95% CI: 1.332–2.001; *P* < 0.001). Other races, AJCC I, grade I, and 2014/2015 diagnosis with HCC were significantly reduce cumulative risk (*P* < 0.05). Similar results were observed from the multivariable Cox analysis.

### PSM method

**Figure 6:**
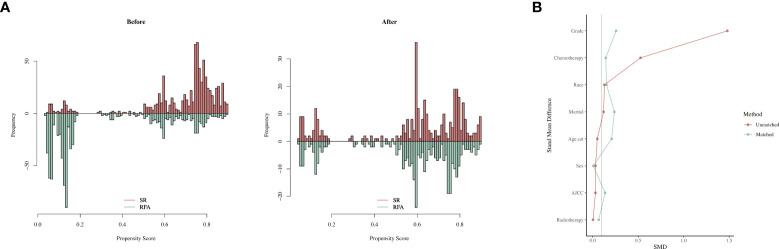
The balance before and after PSM. **(A)** Histogram showing the balance before and after PSM. **(B)** loveplot of PSM.

The propensity score matching (method was subsequently used to balance the baseline characteristics of these two groups). After the above output shows that, when setting the threshold for mean difference to 0.1, all covariates were balanced after PSM. Variables that could influence the outcomes of treatment were included in 1:1 PSM, including “Age.cat,” “Sex,” “Race,” “Marital,” “AJCC,” “Chemotherapy,” “Grade,” “Radiotherapy.” According to the sample composition after PSM sampling matching, SR and SFA data are comparable (**Figure 6**).

### Univariate analysis by CIF after PSM

**Figure 7:**
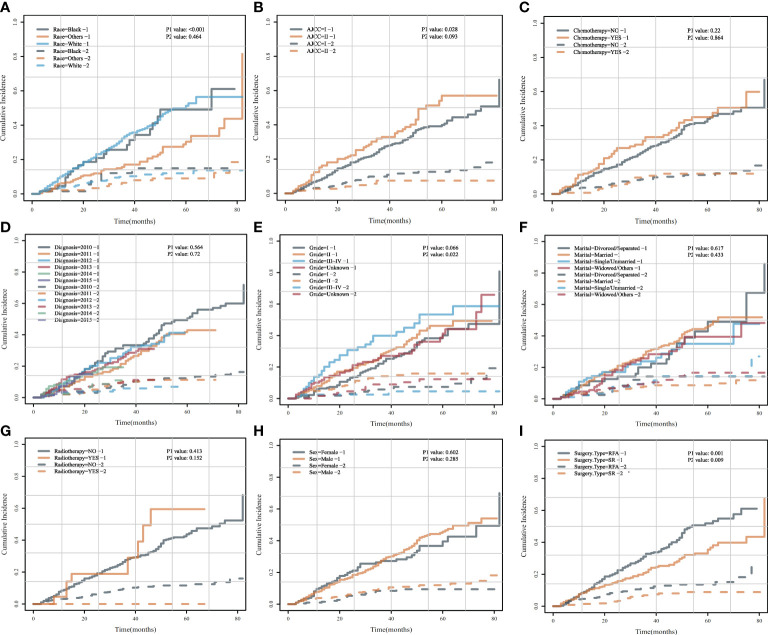
Cumulative incidence curves for each characteristic after PSM.AHCSD/OCSD cumulative incidence curves in HCC patients of different races **(A)**, AJCC **(B)**, chemotherapy **(C)**, diagnosis **(D)**, grade **(E)**, marital **(F)**, radiotherapy **(G)**, gender **(H)**, surgery type **(I)** group with SR or SFA after PSM.

To accurately determine the risk factors of HCSD/OCSD, we carried out univariate analysis by CIF on all the risk factors. CIF curves for all variables were displayed in **Figure 7**. SR was a cumulative risk factor that significantly improved median HCSD/OCSD survival (*P* < 0.05). We also found that other races, AJCC I, and grade 1 were also cumulative risk factors that can significantly increase median survival for HCSD (*P* < 0.05).

### Univariate and multivariable Cox proportional hazard models after PSM

**Figure 8:**
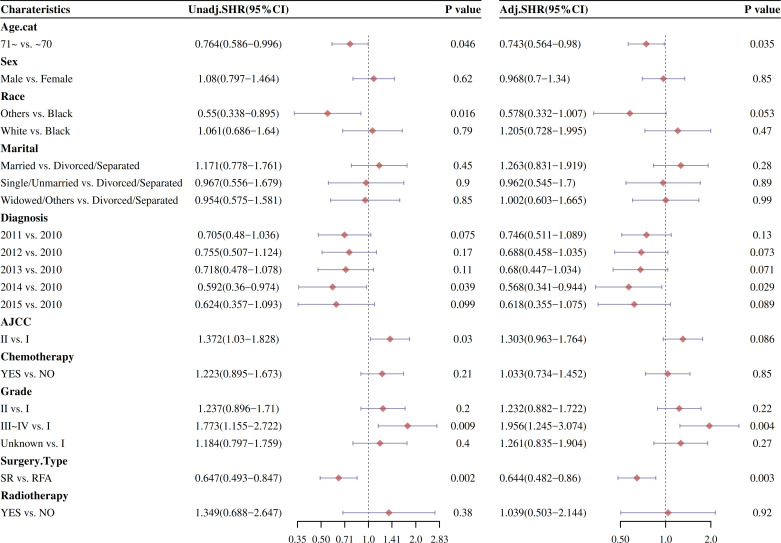
Forestplot of OS and DSS were analyzed by the univariate Cox proportional hazard models after CIF analysis after PSM.

Changes in risk factors of different intervention methods were compared by univariate and multivariate COX regression analysis after PSM (**Figure 8**). After CIF risk competition model calculation and PSM, the univariate and multivariable Cox analysis of factors affecting OS and DSS was analyzed. As showed in **Figure 8**, the univariate analysis indicated that surgery type was significantly positive cumulative incidence factor (HR: 0.647, 95% CI: 0.493–0.847; *P* < 0.001). Other races, AJCC I, and 2014 diagnosis with HCC were significantly reduce cumulative risk (*P* < 0.05).

The effect of stage and treatment modality on OS and DSS was further examined in multivariable Cox proportional hazard models by CIF risk competition model calculation after PSM (**Figure 8**). The multivariable Cox analysis also displayed that surgery type improved median survival of HCC patients (HR: 0.644, 95% CI: 0.482–0.86; *P* < 0.001).

### The survival probability of the radiofrequency therapy group and the conventional surgery group was determined

**Figure 9:**
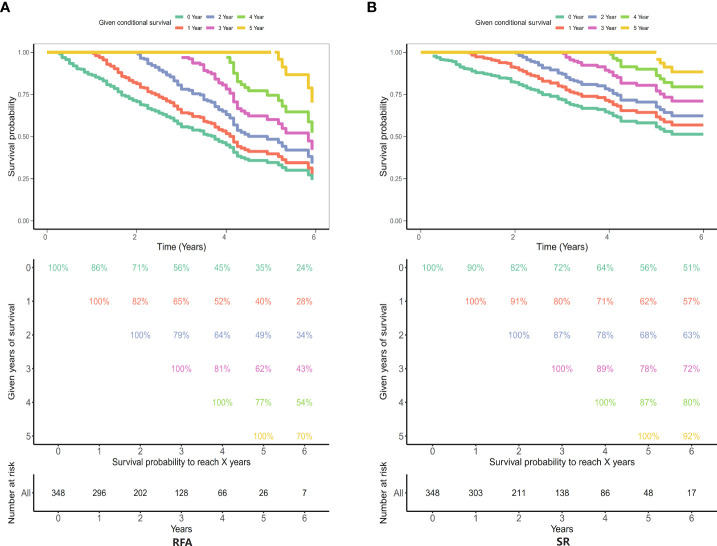
Five-year survival rates in the radiofrequency therapy group and the conventional surgery group. **(A)** Radiofrequency ablation (RFA) group; **(B)** Surgical resection (SR) group.

The conditional disease-specific survival rate of 1–5 years in the conventional surgery group and radiofrequency therapy group. As shown in **Figures 9A, B**, the patient conditional survival was presented with RFA and SR. For RFA, the CS rate at 2 years was about 71% and at 3 years was about 65%. After 5 years of survival, the CS of the patients close to 62%. Similarly, in the SR, the CS of the patients was about 82% after 2 years and about 80% after 3 years of survival. After 5 years of survival, the CS of the patients close to 78%.

## Discussion

The prediction of prognosis is of great significance for treatment choice. Caution is required in the choice of treatment options for elderly people, because they have more comorbidity, and this may have affected postoperative complications and the prognosis. This large population-based study evaluated survival benefits between a balanced cohort of 1,605 patients diagnosed with elderly patients first diagnose for ≤ 5 cm single lesion T1/2 HCC who underwent SR or RFA. Results showed that SR, races, AJCC I, no chemotherapy treatment, and grade I were cumulative risk factors that can significantly improve median survival for HCC. After PSM analysis, only SR was significantly improved median survival of HCC patients when compared with RFA. Comparatively, the 2-, 3-, and 5-year CS rates in SR group is higher than that in the RFA group. Our study indicates that SR is considered the best therapeutic option for elderly patients of < 5 cm single lesion HCC.

Currently, the treatment of the elderly patients with stage T1/2 HCC that isolated lesions are smaller than 5cm is still controversial. Studies have shown that RFA is not superior to hepatectomy for early HCC in terms of tumor recurrence or overall and disease-free survival at 10 years (19). SR is considered the standard treatment option for early HCC patients with liver function preservation (14). Anatomic SR for therapeutic purposes has the advantage of eradicating potential tumor cells from portal vein tributaries (20). However, it may not be suitable for all patients due to the impaired function of background cirrhosis. Our results support that SR treatment can provide survival benefits for elderly patients of < 5 cm single lesion HCC. In theory, RFA may have several advantages over SR (21). Compared with SR, it has lower morbidity and mortality. It also protects liver function better, because less of the non-tumor liver is destroyed (22). Surgical stress and immunosuppression were less common in RFA than in resection (23). Finally, because it is a minimally invasive procedure, RFA may be associated with better quality of life. One possible reason for our result is that RFA is only comparable with hepatic resection in terms of long-term survival for HCC patients with single lesion less than 3cm (5–7), for patients with single lesion 3–5cm, recurrence may be more frequent after RFA than SR, so the effect of SR would be significantly better than RFA.

Our findings agree with the previous study that race, gender, marital status, AJCC stage, chemotherapy, and treatment mode were independent risk factors for the prognosis of elderly patients with HCC (solitary lesions < 5cm) (17). There have been some reports comparing the efficacy of RFA with SR by clinical data or database analysis. In one study, an MVI prediction model using multivariate logistic regression analysis indicated that SR had a lower rate of early recurrence than RFA (*P* < 0.05) (18). Moreover, another study revealed that compared with RFA, LLR laparoscopic hepatectomy ensures a comparable better postoperative condition in elderly patients with solitary HCC (< 3 cm) located in the anterolateral segment despite longer hospital stay and operative time, considering overall survival rates, which was consistent with our results (19). However, a previous study showed that RFA was more effective in patients of primary liver cancer with a diameter of < 5 cm based on biochemical parameters such as alanine aminotransferase (ALT), total bilirubin (TBIL), aspartic aminotransferase (AST), and direct bilirubin (DBIL) compared with those treated with SR (20). The discrepancy of these results may be partly due to improvements in perioperative management of modern surgery and refinements in ablation techniques. Scholars have repeatedly emphasized the standardization of RFA (21). Improper treatment, such as insufficient ablation scope and incomplete ablation process, will greatly increase the short-term recurrence rate.

This study has several limitations of note. First, treatment allocation of retrospective studies is nonrandom and there may be “post screening” differences between experimental and control groups in terms of patient characteristics, so intergroup results in studies are not necessarily due to differences in treatment modalities. Therefore, we applied PSM method. After CIF risk competition model calculation and PSM, the patients who received SR still had better results than those who received RFA. This provided strong evidence that our conclusions were robust. Second, we did not carry out the subgroup analysis for < 3 cm and 3–5cm, this may be a potential confounding factor, which need to be addressed in the further studies. Third, because RFA is a technique-dependent procedure, the SEER database includes too many heterogeneities, such as technology and equipment. In addition, based on hospital data, it is easy to have defects such as patient selection bias and small sample size. Fourth, in the public SEER data set, the variables of performance status, comorbidities, and hepatic reserves such as Child-Pugh score and ALBI grade, and background liver disease were not available.

We can only include the existing variables as much as possible to fully adjust the confounding factors, and through multifactor analysis, PSM, and other methods to fully adjust the confounding, but some known or unknown confounding is indeed inevitable in observational research.

In conclusion, SR has significant advantage over RFA in elderly patients with first diagnosed stages I and II HCC (single nodular HCCs ≤ 5 cm). The present data may contribute to develop a more suitable treatment strategy for the elderly patients of with early stage HCC.

## Data availability statement

The original contributions presented in the study are included in the article/supplementary material. Further inquiries can be directed to the corresponding author.

## Author contributions

HS: Conceptualization; Data selection; Data analysis; Project administration. QX: Writing—original draft; Writing—review and editing. FT: Conceptualization; Data analysis; Writing—original draft. YY: Conceptualization; Writing—original draft. BQ: Data selection; Data analysis. PX: Data selection; Data analysis. All authors contributed to the article and approved the submitted version.

## Funding

This work was supported by Health Commission of Hunan Province (20201513) and Changsha Natural Science fund (kq2014213); the self-funded research project of Guangxi Zhuang Autonomous Region health and Family Planning Commission (Z20200093).

## Conflict of interest

The authors declare that the research was conducted in the absence of any commercial or financial relationships that could be construed as a potential conflict of interest.

## Publisher’s note

All claims expressed in this article are solely those of the authors and do not necessarily represent those of their affiliated organizations, or those of the publisher, the editors and the reviewers. Any product that may be evaluated in this article, or claim that may be made by its manufacturer, is not guaranteed or endorsed by the publisher.
